# HLA-G polymorphism impacts the outcome of oral HPV infections in women

**DOI:** 10.1186/s12879-021-06079-7

**Published:** 2021-05-04

**Authors:** Anna Jaakola, Michel Roger, Marie-Claude Faucher, Kari Syrjänen, Seija Grénman, Stina Syrjänen, Karolina Louvanto

**Affiliations:** 1 Turku University Hospital, University of Turku, Turku, Finland; 2 Department of Obstetrics and Gynecology, Kymenlaakso Central Hospital, Kotkantie 41, 48210 Kotka, Finland; 3 Centre de Recherche du CHUM, Montreal, Canada; 4 Département de Microbiologie, Infectiologie et Immunologie,de l’Université de Montréal, Montreal, Canada; 5 Department of Clinical Research, Biohit Oyj, Helsinki, Finland; 6 Department of Oral Pathology and Radiology, University of Turku, Turku, Finland; 7 Department of Pathology, Turku University Hospital, University of Turku, Turku, Finland; 8 Department of Obstetrics and Gynecology, Tampere University and Tampere University Hospital, Tampere, Finland

**Keywords:** Human papilloma virus, HPV, HLA-G, Genotype, Oral, Infection

## Abstract

**Backround:**

Human leukocyte antigen (HLA)-G may have an important role in the natural history of human papillomavirus (HPV) infection. Our aim was to evaluate the role of HLA-G in the outcome of genital and oral HPV infections in women.

**Methods:**

Analyses included 306 women from the Finnish Family HPV-study and were followed-up for six years. Genital and oral samples were tested for 24 different HPV types with multiplex HPV genotyping. HLA-G alleles were determined through direct DNA-sequencing. Unconditional logistic regression was used to determine the associations between HLA-G genotypes and HPV infection outcomes.

**Results:**

Ten HLA-G alleles were identified. Most common HLA-G genotypes were the wild type G*01:01:01/01:01:01 (31.3%) followed by G*01:01:01/01:01:02 (26.8%). G*01:01:01/01:01:01 genotype was associated with increased risk of oral HPV infections by any HPV type or single-type with OR = 1.86 (95% CI 1.14–3.04, *P* = 0.01) and 2.22 (95% CI 1.14–3.71, *P* = 0.02), respectively. G*04:01+ allele and the G*01:01:01/01:04:01 genotype both protected from any and single oral HPV infections; OR = 0.46 (95% CI 0.23–0.89, P = 0.02) and 0.53 (95% CI 0.23–0.97, *P* = 0.03), respectively. G*01:01:02/01:04:01 genotype increased significantly the risk of infertility and its treatments, with respective OR = 5.06 (95% CI 1.22–21.02, P = 0.03) and OR = 9.07 (95% CI 1.22–39.50, P = 0.03). Both HLA-G alleles and genotypes showed several significant associations with the outcomes of oral HPV infections, but none of them had any impact on the outcomes of genital HPV infections in these women.

**Conclusions:**

The host HLA-G genotypes appear to impact the outcomes of oral HPV infections in women but have little if any effect on genital HPV status or infection outcomes.

## Backround

Human Papillomavirus (HPV) infections are highly common, and it is estimated that 80% of sexually active women contract a HPV-infection during their lifetime [1–3] . Persistent genital infection with high-risk (HR) HPV is shown to be involved in nearly all cervical cancers and its precursors [4–7]. Increasing evidence also implicates that HPV infections play a significant role in the etiology of head and neck carcinomas, oropharyngeal cancer in particular [8, 9]. Globally, HPV infections are currently associated up to 4,5% of all new cancer cases and among women 8,6% new cancer cases are HPV induced [5, 7].

Only a small percentage of HPV infections progress to cervical cancer (CC). Impaired reactivity of the cell-mediated immunity (CMI) and the human leukocyte antigen (HLA) system to viral antigens seem to increase the risk of CC [10, 11]. The vast majority of the previous studies have focused on the association between HLA class II alleles and cervical carcinoma [11–15], but data are emerging to implicate an association between HLA-G polymorphisms and the natural history of cervical HPV infections (prevalence and persistence) as well as in cervical and oral carcinomas [11–15]. Human leukocyte antigen (HLA)-G is a non-classical HLA class Ib molecule, first identified to be present in placental cells of fetal origin and playing a role in immune tolerance during pregnancy [16–18]. However, HLA-G can be expressed de novo at high levels in several pathological conditions, including some tumors as well as during microbial or viral infections, leading to the impairment of the immune response against tumor cells or infectious pathogens, respectively [11, 19]. HLA-G has been found to play an important role in several aspects of the female reproductive health [12, 20–24].

In the present study, our aim was to evaluate the potential impact of HLA-G polymorphism in the outcomes of genital and oral HPV infections among women as well as the HLA-G role in different characteristics of the female reproductive health.

## Methods

### Finnish family HPV-study

The present study is part of the Finnish Family HPV (FFHPV) cohort study, which was initiated in 1998 to investigate the dynamics of HPV transmission between the family members [25, 26]. At baseline, 329 pregnant mothers, 171 fathers and 331 their newborn babies were recruited. The present study focus only on women followed at 3rd trimester (>36 gestational weeks), 2-, 6-,12-,24-,36- and 72-months’ visits (between the years 1998 to 2006) as described earlier [25]. All the families are of Caucasian origin (native Finnish population). At the baseline visit, all women were pregnant (>36 gestational weeks), with the mean age of 25.6 years (SD ± 0.2), range 18–46 years. Detailed data were collected by structured questionnaire at baseline, complemented with the information about the delivery as well as the child’s anthropometrics. The Research Ethics Committee of Turku University Hospital (#3/1998 and #2/2006) has approved the study protocol and its amendments. All the participants gave their written consent for the cohort study.

### Samples

Oral and genital scrapings from all women were collected for HPV-testing with a cytobrush (MedScand, Malmö, Sweden) at baseline and during the follow-up visits at 2, 6, 12, 24, 36 and 72 months [25]. HPV was detected with PCR, using My09/My11 and GP05+/GP06+ − primers, as previously described in detail [27]. Samples were all tested with Luminex-based Multimerix kit, which detects 24 low-risk (LR)- and high-risk (HR)- HPV genotypes (LR-HPV: 6, 11, 42, 43, 44 and HR-HPV: 16, 18, 26, 31, 33, 35, 39, 45, 51, 52, 53, 56, 58, 59, 66, 68, 70, 73, 82. Genomic DNA for HLA-G typing was isolated form frozen blood cells using MagNA Pure 96 System (Roche). HLA-G alleles were determined by direct DNA-sequencing of the nucleotide regions encompassing the HLA-G exons 2–4 (1718 bp) as previously described [12].

### Statistical analysis

This study includes 306 women of the FFHPV-Study. Stata 15.0 (Stata Corp., College Station, TX) was used for all statistical analyses, performed two-sided and declared significant at the *P*-value < 0.05 level. Women with no HLA-G genotyping result available (*n* = 23/329) were excluded from the analyses. Woman was classified as HPV positive when any of the samples taken during the 72 months follow-up was HPV positive (any type as single or multiple infection. Single infection defined as always only one HPV type present at the follow-up visits and multiple HPV infection as two or more HPV types presented simultaneously during the follow up visits. In statistical analysis, we only considered HLA alleles and genotypes that were present in at least in 3% of the study group. Low resolution groups were generated for the following alleles Group 01:01: *010101, *010102, *010103, *010114 and Group 04:01: *01040 and *010404.

Unconditional logistic regression analysis was used to determine the associations between HLA-G alleles or genotypes and the genital and oral HPV-infection outcomes as well as their role as risk factors of several reproductive health endpoints. An incident HPV-infection was recorded when a baseline HPV-negative woman tested HPV-positive at any of the follow-up visits. HPV clearance denotes all cases where a previously HPV-positive women tested HPV-negative at any follow-up. visits and remained HPV-negative until the end of the follow-up. Persistent HPV infection was defined when HPV positivity was recorded in two or more consecutive visits during the follow-up.

## Results

**Fig. 1:**
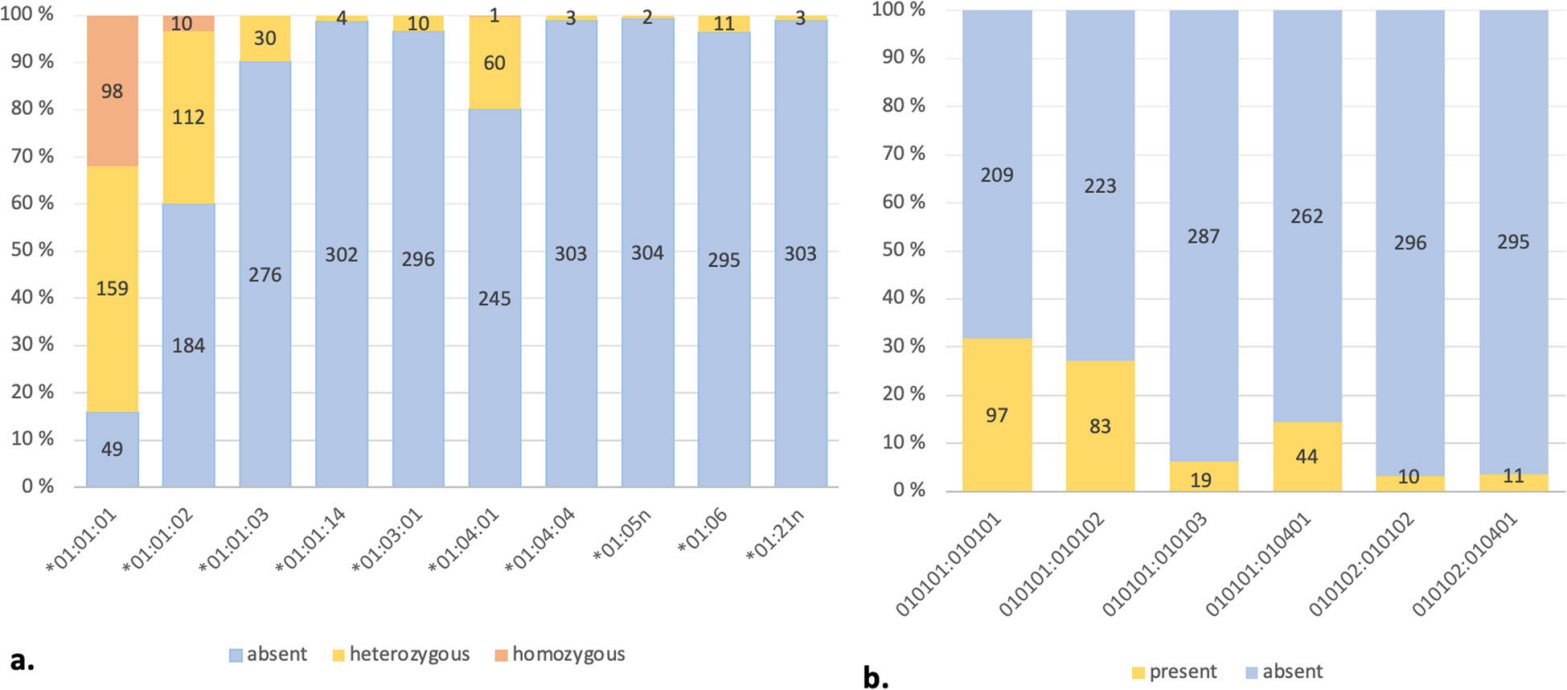
Prevalence of different HLA-G a. alleles and b. genotypes among 306 women in the Finnish Family HPV-study. Only alleles and genotypes with > 3% prevalence among these women are shown

Ten different HLA-G alleles and 24 different genotype combinations were were identified among the women of the FFHPV study. HLA-G alleles and genotypes with > 3% prevalence are shown in Fig. 1. The most common HLA-G allele was the wild-type *01:01:01 of which 32% (*n* = 98) of the women being homozygous, 52% (*n* = 159) heterozygous and only 16% (*n* = 49) women missing this allele. Of the HLA-G genotypes, *01:01:01/01:01:01 (31.3%) was the most prevalent, followed by G*01:01:01/01:01:02 (26.8%).

HLA-G alleles and genotypes as related to genital and oral HPV-prevalence (single and multiple-type) are depicted in Table 1**.** No significant associations were observed between HLA-G alleles/genotypes and genital HPV infections. As to oral HPV infections, allele G*01:01:03 and genotype G* 01:01:01/01:01:03 were associated with increased risk of multiple-type oral HPV infections during the follow up, OR = 3.32 (95% CI 1.18–9.34, *P* = 0.02) and OR = 4.44 (95% CI 1.39–14.20, *P* = 0.01), respectively. G*01:01:01/01:04:01 genotype and the G’01:04+ group both protected from any and single-type oral HPV infections; OR = 0.46 (95% CI 0.23–0.89, *P* = 0.02) and OR = 0.53 (95% CI 0.30–0.94, *P* = 0.03), respectively G*01:01:01/01:01:01 genotype seemed to predispose the women to any HPV type or single-type infection with OR = 1.86 (95% CI 1.14–3.04, *P* = 0.01) and OR = 2.22 (95% CI 1.14–3.71, P = 0.02), respectively.

**Table 1:** HLA-G alleles, low-resolution groups and genotypes, as related to genital and oral HPV status^1^ of the women

	Genital			Oral		
HLA-G	Any HPV (*n* = 250)	Single (*n* = 119)	Multiple (2+) (*n* = 131)	Any HPV (*n* = 154)	Single (*n* = 120)	Multiple (2+) (*n* = 34)
Alleles						
*01:01:01	1.01 (0.46–2.21)	0.84 (0.36–1.98)	1.20 (0.50–2.87)	0.88 (0.48–1.62)	1.01 (0.52–1.97)	0.58 (0.23–1.44)
*01:01:02	0.94 (0.52–1.70)	0.90 (0.47–1.73)	0.97 (0.52–1.84)	0.83 (0.53–1.31)	0.83 (0.51–1.35)	0.85 (0.40–1.83)
*01:01:03	0.88 (0.34–2.28)	0.76 (0.26–2.22)	1.00 (0.36–2.74)	1.80 (0.83–3.93)	1.42 (0.61–3.35)	**3.32 (1.18–9.34)**
*01:03:01	0.89 (0.18–4.32)	0.94 (0.17–5.29)	0.85 (0.15–4.78)	0.24 (0.05–1.13)	0.15 (0.02–1.23)	0.55 (0.07–4.51)
*01:04:01	1.02 (0.49–2.12)	1.03 (0.47–2.29)	1.01 (0.46–2.22)	0.57 (0.32–1.02)	0.55 (0.29–1.02)	0.67 (0.26–1.73)
*01:06	2.29 (0.29–18.28)	3.44 (0.41–28.64)	1.29 (0.13–12.67)	1.76 (0.51–6.15)	1.28 (0.31–5.21)	3.58 (0.76–16.81)
Groups^2^						
G01:01	1.12 (0.12–10.20)	1.06 (0.09–11.98)	1.17 (0.10–13.20)	0.67 (0.11–4.07)	0.79 (0.11–5.67)	0.44 (0.04–5.00)
G04:01	1.07 (0.52–2.22)	1.09 (0.49–2.40)	1.06 (0.49–2.32)	**0.53 (0.30–0.94)**	**0.51 (0.28–0.95)**	0.62 (0.24–1.61)
Genotypes						
010101:010101	0.80 (0.44–1.47)	0.84 (0.43–1.65)	0.76 (0.39–1.48)	**1.86 (1.14–3.04)**	**2.22 (1.32–3.71)**	0.92 (0.39–2.21)
010101:010102	0.82 (0.44–1.55)	0.71 (0.35–1.44)	0.94 (0.47–1.86)	0.73 (0.44–1.21)	0.80 (0.47–1.37)	0.49 (0.19–1.27)
010101:010103	1.97 (0.44–8.78)	1.95 (0.40–9.48)	1.99 (0.42–9.53)	1.75 (0.67–4.57)	1.09 (0.36–3.33)	**4.44 (1.39–14.20)**
010101:010401	1.01 (0.44–2.31)	0.93 (0.37–1.33)	1.08 (0.45–2.62)	**0.46 (0.23–0.89)**	**0.47 (0.23–0.97)**	0.41 (0.12–1.44)
010102:010102	2.05 (0.25–16.55)	2.92 (0.43–24.86)	1.29 (0.13–12.67)	1.50 (0.41–5.42)	1.28 (0.31–5.21)	2.31 (0.41–13.17)
010102:010401	2.29 (0.29–18.28)	2.92 (0.34–24.86)	1.73 (0.19–15.85)	1.19 (0.36–3.99)	1.01 (0.27–3.86)	1.84 (0.34–9.90)

^1^HPV positivity taken as positive for any HPV type during the 72 months follow-up (part of single or multiple infection), Single infection: always only one HPV type present at the follow-up visits and multiple: two or more HPV types presented during the follow up visits. ^2^Groups as low resolution for the following alleles G01:01: *010101, *010102, *010103, *010114 and G04:01: *01040 and *010404. Only those HLA-G alleles and genotypes that where > 3% prevalent among the women where included to the analyses. The significant associations are bolded

HLA-G alleles, groups and genotypes as related to genital and oral HPV infection outcomes (always negative, incidence, clearance and persistence) are shown in Table 2**.** No significant association was detected between the genital HPV-infection outcomes and HLA-G alleles or genotypes. As to oral HPV infections**,** allele G*01:01:02 was associated with decreased clearance of oral HPV infection OR = 0.50 (95% CI 0.29–0.79, *P* = 0.03) and the genotype G*01:01:02/01:04:01 increased the risk of oral persistence, with OR = 4.01 (95% CI 1.19–13.53, P = 0.03). HLA Group G04:01+ decreased the risk of incident oral HPV by 2-fold; OR = 0.51 (95% CI 0.27–0.98, P = 0.03) and increased the probability of women remaining HPV-negative throughout the follow-up; OR = 1.87 (95% CI 1.06–3.30). Similar association with always HPV-negative status was also found for genotype G*01:01:02/01:04:01, with OR = 2.18 (95% CI 1.12–4.26, *P* = 0.02). Genotype G*01:01:01/01:01:01 was related to increased clearance of any oral HPV infection but also decreased the probability of always HPV-negative status with OR = 1.18 (95% CI 1–28-3.71, *P* = 0.01) and OR = 0.54 (95% CI 0.33–0.88, P = 0.02), respectively (Table 2).

**Table 2:** HLA-G alleles, low-resolution groups and genotypes as related to outcomes of the genital and oral HPV infections during the six-year follow-up

	Genital HPV infection outcomes^1^				Oral HPV infection outcomes^1^			
HLA-G	Always negative (*n* = 106)	Incidence (*n* = 200)	Clearance (*n* = 57)	Persistence (*n* = 143)	Always negative (*n* = 205)	Incidence (*n* = 101)	Clearance (*n* = 27)	Persistence (*n* = 74)
Alleles	OR (95% CI)				OR (95% CI)			
*01:01:01	0.99 (0.45–2.19)	0.72 (0.37–1.40)	1.14 (0.61–2.12)	0.81 (0.44–1.50)	1.14 (0.62–2.10)	1.14 (0.59–2.21)	1.92 (0.86–4.29)	0.68 (0.34–1.32)
*01:01:02	1.06 (0.59–1.92)	1.08 (0.67–1.75)	0.88 (0.55–1.40)	1.06 (0.67–1.67)	1.20 (0.76–1.90)	0.87 (0.53–1.42)	**0.50 (0.29–0.79)**	0.96 (0.56–1.65)
*01:01:03	1.13 (0.44–2.91)	1.26 (0.56–2.87)	1.18 (0.54–2.64)	1.34 (0.63–2.85)	0.55 (0.25–1.21)	1.90 (0.89–4.07)	1.07 (0.46–2.51)	1.16 (0.49–2.72)
*01:03:01	1.12 (0.23–5.42)	0.79 (0.22–2.86)	1.14 (0.32–4.13)	2.75 (0.70–10.82)	4.22 (0.88–20.22)	0.50 (0.10–2.39)	NC	0.34 (0.04–2.72)
*01:04:01	0.98 (0.47–2.03)	0.78 (0.44–1.38)	1.08 (0.61–1.91)	0.81 (0.46–1.43)	1.74 (0.98–3.09)	0.54 (0.28–1.04)	0.66 (0.33–1.33)	0.92 (0.47–1.78)
*01:06	0.44 (0.05–3.48)	2.45 (0.52–11.55)	1.34 (0.38–4.68)	0.64 (0.18–2.24)	0.57 (0.16–1.98)	1.17 (0.33–4.08)	1.10 (0.28–4.25)	1.84 (0.52–6.46)
Groups^2^								
G01:01	0.89 (0.10–8.16)	1.26 (0.21–7.68)	0.32 (0.04–2.94)	0.58 (0.95–3.52)	1.49 (0.25–9.05)	0.74 (0.12–4.47)	1.38 (0.15–12.49)	0.47 (0.77–2.88)
G04:01	0.93 (0.45–1.92)	8.83 (0.47–1.47)	1.06 (0.60–1.87)	0.89 (0.51–1.55)	**1.87 (1.06–3.30)**	**0.51 (0.27–0.98)**	0.63 (0.32–1.26)	0.87 (0.45–1.69)
Genotypes								
010101:010101	1.25 (0.68–2.29)	1.19 (0.71–1.99)	1.01 (0.61–1.65)	0.87 (0.54–1.41)	**0.54 (0.33–0.88)**	1.49 (0.90–2.47)	**1.18 (1–28-3.71)**	1.44 (0.83–2.49)
010101:010102	1.22 (0.64–2.29)	0.79 (0.47–1.33)	1.09 (0.66–1.84)	0.89 (0.54–1.47)	1.37 (0.83–2.28)	0.90 (0.52–1.55)	0.62 (0.33–1.51)	0.75 (0.41–1.38)
010101:010103	0.51 (0.11–2.26)	2.99 (0.85–10.49)	1.09 (0.40–2.94)	1.61 (0.63–4.13)	0.57 (0.22–1.49)	1.91 (0.75–4.85)	1.05 (0.36–3.01)	1.49 (0.54–4.06)
010101:010401	0.99 (0.43–2.27)	0.59 (0.31–1.12)	0.95 (0.50–1.82)	0.68 (0.35–1.31)	**2.18 (1.12–4.26)**	0.64 (0.31–1.32)	0.97 (0.46–2.03)	0.45 (0.18–1.11)
010102:010102	0.49 (0.06–3.92)	1.25 (0.32–4.92)	0.60 (0.16–2.27)	1.14 (0.32–4.04)	0.67 (0.18–2.41)	0.87 (0.22–3.42)	1.26 (0.32–5.01)	1.36 (0.34–5.39)
010102:010401	0.44 (0.05–3.48)	5.53 (0.70–43.77)	0.91 (0.27–3.04)	1.38 (0.41–4.63)	0.84 (0.25–2.81)	0.20 (0.02–1.54)	NC	**4.01 (1.19–13.53)**

^1^HPV outcomes definitions: incidence: baseline negative and acquired a HPV infection during follow-up, clearance: HPV positive and cleared the HPV (staying HPV negative to the end of the follow-up): persistence: recoded two or more consequence visit as HPV positive during the follow-up. ^2^Groups as low resolution for the following alleles G01:01: *010101, *010102, *010103, *010114 and G04:01: *01040 and *010404. Only those HLA-G alleles and genotypes that where > 3% prevalent among the women where included to the analyses. NC: Not Computable. The significant associations are bolded

We also investigated the associations between HLA-G alleles and genotypes with reproductive risk factors of HPV infection recorded in these women at baseline and during the follow-up (Table 3 and 4**)**. At the HLA-G allele level only allele G*01:01:02 showed associated with increased risk of having atopic tendency, OR = 1.9 (95% CI 1.00–3.73, *P* = 0.05) while allele *01:06 increased the risk having an inducted labour, OR = 6.48 (95% CI 1.28–32.77, *P* = 0.02) (Table 3). In HLA-G allele groups, G*01:01- group was associated with decreased risk of having an early labor (< 37 week), OR = 0.07 (95%CI 0.01–0.72, *P* = 0.03). This trend was also seen in the high resolution wild type alleles, but it did not, P = 0. reach statistical significance. At the genotype level (Table 4), HLA-G*01:01:02/01:04:01 was associated with an increased infertility and the recorded treatments, with respective OR = 5.06 (95%CI 1.22–21.02, *P* = 0.03) and OR = 9.07 (95% CI 1.22–39.50, P = 0.03). Genotype G*01:01:02/01:04:01 was shown to decrease the likelihood of skin wart history during follow-up, OR = 0.18 (95%CI 0.04–0.87, *p* = 0.03). Self-reported oral warts were associated with genotypes G*01:01:01/01:01:02 and G*01:01:01/01:01:03, with OR = 4.85 (95% CI 1.13–20.85, P = 0.03) and OR = 6.00 (95% CI 1.11–32.46, *P* = 0.04), respectively. In addition, HLA-G*01:01:01/01:01:03 genotype showed a significant association with an abnormal placenta, OR = 5.51 (95% CI 1.03–29.37, *P* = 0.05).

**Table 3:** Association between HLA-G alleles as their low-resolution groups and risk factors for HPV infection recorded by the baseline questionnaire

Risk factors	HLA-G alleles OR (95%Cl)						HLA-G groups OR (95%Cl)	
	*01:01:01	*01:01:02	*01:01:03	*01:03:01	*01:04:01	*01:06	G01:01	G04:01
Miscarriages (≥1) (*n* = 39)	0.56 (0.25–1.29)	1.67 (0.83–3.35)	1.51 (0.53–4.28)	2.87 (0.71–11.65)	0.59 (0.22–1.60)	0.63 (0.08–5.03)	0.62 (0.07–5.74)	0.56 (0.21–1.52)
Infertility (*n* = 24)	0.44 (0.17–1.13)	2.28 (0.97–5.34)	0.38 (0.05–2.95)	1.19 (0.14–9.80)	0.78 (0.26–2.38)	NC	NC	0.74 (0.24–2.27)
Infertility treatments (n = 15)	0.37 (0.12–1.15)	2.34 (0.81–6.77)	0.65 (0.08–5.11)	NC	2.11 (0.69–6.45)	NC	0.22 (0.02–2.08)	2.01 (0.66–6.13)
Sexually transmitted diseases (HSV, Chlamydia) (*n* = 63)	1.12 (0.51–2.47)	0.62 (0.34–1.13)	1.00 (0.39–2.58)	0.44 (0.54–3.52)	0.75 (0.35–1.57)	0.89 (0.19–4.22)	0.37 (0.06–2.24)	0.82 (0.40–1.68)
Self reported warts								
Genital (*n* = 80)	0.92 (0.45–1.87)	1.08 (0.63–1.84)	0.67 (0.26–1.72)	1.10 (0.28–4.35)	1.85 (0.99–3.46)	0.56 (0.12–2.63)	0.39 (0.05–2.79)	**1.91 (1.03–3.53)**
Oral (*n* = 8)	1.35 (0.16–11.25)	4.82 (0.96–24.36)	3.21 (0.62–16.77)	4.07 (0.45–36.75)	NC	NC	NC	NC
Skin (*n* = 166)	1.35 (0.73–2.50)	0.90 (0.57–1.42)	1.86 (0.84–4.12)	0.57 (0.16–2.08)	0.59 (0.33–1.03)	4.12 (0.88–19.39)	0.76 (0.12–4.59)	0.64 (0.37–1.12)
Allergies (*n* = 126)	0.98 (0.51–1.87)	0.94 (0.58–1.52)	0.74 (0.33–1.70)	0.29 (0.06–1.41)	1.35 (0.75–2.43)	2.21 (0.63–7.73)	1.23 (0.20–7.48)	1.35 (0.75–2.42)
Atopia (*n* = 43)	0.51 (0.23–1.11)	**1.9 (1.00–3.73)**	1.23 (0.44–3.45)	0.58 (0.07–4.69)	0.93 (0.40–2.14)	1.19 (0.25–5.69)	NC	1.23 (0.57–2.69)
Vulvovaginitis (*n* = 48)	0.56 (0.11–2.87)	0.94 (0.22–4.02)	NC	4.10 (0.45–36.89)	NC	NC	NC	NC
Menarche								
Late (>14 years) (*n* = 21)	1.12 (0.31–3.99)	0.81 (0.31–2.10)	1.69 (0.46–6.20)	1.55 (0.18–13.02)	0.97 (0.31–3.01)	NC	NC	1.25 (0.44–3.62)
Early (<11 years) (n = 7)	1.18 (0.14–10.04)	0.60 (0.11–3.17)	NC	NC	1.57 (0.30–8.32)	4.1 (0.45–37.35)	NC	1.50 (0.28–7.93)
Start of labour								
Own contractions (*n* = 145)	1.33 (0.69–2.59)	0.83 (0.51–1.36)	0.52 (0.23–1.19)	1.19 (0.31–4.54)	1.07 (0.59–1.95)	2.93 (0.58–14.79)	0.35 (0.04–3.37)	1.02 (0.57–1.85)
Rupture of the membranes (*n* = 52)	1.15 (0.48–2.76)	1.22 (0.66–2.29)	0.33 (0.75–1.43)	0.55 (0.07–4.48)	0.59 (0.25–1.40)	NC	0.67 (0.07–6.58)	0.57 (0.24–1.36)
Induction (n = 52)	1.28 (0.62–2.63)	0.70 (0.42–1.19)	0.84 (0.35–2.01)	1.66 (0.43–6.33)	1.47 (0.80–2.72)	**6.48 (1.28–32.77)**	0.49 (0.07–3.51)	1.42 (0.77–2.62)
Labour weeks								
Early (<37 weeks) (n = 8)	0.95 (0.11–8.33)	0.75 (0.14–4.16)	NC	NC	0.8 (0.09–6.98)	5.8 (0.62–54.35)	**0.07 (0.01–0.72)**	0.77 (0.09–6.69)
Late (>40 weeks) (*n* = 181)	0.94 (0.51–1.74)	1.05 (0.66–1.66)	1.96 (0.87–4.43)	1.19 (0.33–4.31)	1.17 (0.66–2.07)	0.44 (0.13–1.53)	1.92 (0.32–11.66)	1.06 (0.61–1.87)
Abnormal placenta (n = 9)	NC	0.49 (0.10–2.49)	3.21 (0.62–16.69)	NC	0.57 (0.07–4.69)	NC	NC	0.54 (0.07–4.50)

The significant associations are bolded

**Table 4:** Association between HLA-G genotypes and risk factors for HPV infection recorded by the baseline questionnaire

Risk factors	HLA-G genotype OR (95% CI)					
	*010101:010101	*010101:010102	*010101:010103	*010101:010401	*010102:010102	*010102:010401
Miscarriages (≥1) (n = 39)	0.81 (0.37–1.75)	1.13 (0.53–2.42)	1.39 (0.38–5.10)	0.50 (0.15–1.73)	2.18 (0.42–11.24)	0.70 (0.09–5.67)
Infertility (n = 24)	1.09 (0.45–2.66)	1.13 (0.45–2.85)	0.70 (0.09–5.51)	0.24 (0.03–1.84)	1.54 (0.18–13.07)	**5.06 (1.22–21.02)**
Infertility treatments (n = 15)	0.54 (0.15–1.95)	1.36 (0.45–4.13)	1.17 (0.14–9.52)	0.42 (0.05–3.28)	NC	**9.07 (2.08–39.50)**
Sexually transmitted diseases (n = 63)	1.40 (0.78–2.51)	0.68 (0.35–1.33)	1.08 (0.34–3.37)	0.59 (0.24–1.48)	0.44 (0.05–3.52)	0.89 (0.19–4.22)
Warts						
Genital (n = 80)	0.62 (0.34–1.12)	1.18 (0.66–2.11)	0.53 (0.15–1.89)	1.88 (0.94–3.77)	0.85 (0.17–4.29)	0.72 (0.15–3.55)
Oral (n = 8)	NC	**4.85 (1.13–20.85)**	**6.00 (1.11–32.46)**	NC	NC	NC
Skin (n = 166)	0.96 (0.59–1.56)	1.29 (0.77–2.14)	1.98 (0.73–5.35)	0.69 (0.37–1.32)	0.87 (0.25–3.08)	**0.18 (0.04–0.87)**
Allergies (n = 126)	1.07 (0.64–1.77)	0.88 (0.52–1.50)	0.95 (0.34–2.63)	1.13 (0.58–2.20)	2.08 (0.49–8.89)	2.52 (0.62–10.29)
Atopia (n = 43)	0.62 (0.29–1.32)	1.38 (0.68–2.79)	0.34 (0.04–2.63)	0.59 (0.20–1.74)	1.80 (0.35–9.25)	2.78 (0.67–11.55)
Vulvovaginitis (n = 48)	1.31 (0.31–5.60)	0.39 (0.05–3.20)	NC	NC	NC	NC
Menarche						
Late (>14 years) (n = 21)	0.96 (0.36–2.59)	0.67 (0.22–2.08)	3.12 (0.81–12.03)	2.37 (0.44–12.66)	NC	1.37 (0.16–11.38)
Early (<11 years) (n = 7)	1.71 (0.37–7.83)	0.45 (0.05–3.82)	NC	1.01 (0.28–3.64)	NC	NC
Start of labour						
Own contractions (n = 145)	1.11 (0.66–1.87)	0.96 (0.57–1.65)	0.65 (0.24–1.75)	1.12 (0.57–2.18)	0.75 (0.20–2.86)	0.75 (0.20–2.86)
Rupture of the membranes (n = 52)	1.43 (0.75–2.74)	1.31 (0.67–2.55)	0.26 (0.03–2.03)	0.43 (0.15–1.28)	1.28 (0.26–6.37)	1.28 (0.26–6.37)
Induction (*n* = 91)	0.87 (0.50–1.53)	0.79 (0.44–1.41)	1.12 (0.40–3.13)	1.74 (0.89–3.44)	0.57 (0.12–2.81)	0.72 (0.81–4.07)
Labour weeks						
Early (<37 weeks) (n = 8)	1.08 (0.19–5.99)	2.75 (0.54–13.90)	NC	NC	NC	NC
Late (>40 weeks) (n = 181)	0.82 (0.51–1.33)	0.97 (0.59–1.62)	2.32 (0.81–6.61)	1.30 (0.68–2.50)	0.51 (0.14–1.86)	0.95 (0.28–3.17)
Abnormal placenta (n = 9)	1.30 (0.30–5.56)	0.89 (0.18.4.51)	**5.51 (1.03–29.37)**	0.85 (0.10–7.06)	NC	NC

The significant associations are bolded

## Discussion

In the present study, we identified ten different HLA-G alleles and 24 different genotype combinations among the 306 women of the FFHPV-Study. We found an association with certain HLA-G alleles and genotypes with the outcomes of oral HPV-infections, but interesting enough no such associations with the outcomes of genital HPV-infections. Additionally, certain HLA-G alleles and genotypes seemed to be linked with some characteristics of the women’s reproductive health.

So far there are only a few studies available investigating the impact of HLA-G polymorphism in the natural history of HPV-infections. Most of these investigations are evaluating the association between HLA-G and HPV-induced cervical premalignant and malignant lesions [12, 13, 21–24]. The data on HLA-G polymorphism in oral HPV-infections are, to our knowledge, lacking. So far, these studies have evaluated malignant lesions of the oropharynx, where HLA-G polymorphism have been studied, but only using immunohistochemistry in the biopsy samples with mono−/or polyclonal antibodies for HLA-G or assessing the polymorphic sites at 5′URR (upstream regulatory region) and 3′UTR (untranslated region) by polyacrylamide gel electrophoresis (PAGE) [22–24]. In our study none of the women developed any premalignant or malignant oral lesions during the six-year follow-up. Oral mucosa was carefully examined at the last visit [28].

Our results revealed that homozygous wildtype genotype G*01:01:01/01:01:01 showed to increase both the clearance of any oral HPV infection (OR = 1.18) (Table 2) and having any oral HPV-infections during the follow-up (OR = 1.86) (Table 1). However, this wild-type genotype did not impact on the HPV persistence that would be mandatory for the progression of an asymptomatic infection to a HPV induced premalignant lesion (i.e. oral potentially malignant lesion). A similar protective effect for oral HPV infection was seen with G*04:01+ (OR 1.87) as with genotype G*01:01:01/01:04:01 (OR 2.18). There are no previous studies on the oral mucosa with the HLA-G wildtype, but a few regarding the genital mucosa [12, 13, 23]. Of these studies only Metcalfe and coworkers showed that allele G*01:01:01(OR = 2.23) and heterozygous genotype with G*01:01:01 (OR = 2.14) were increasing both the risks of genital LR-HPV infection and genital multiple-type infections versus single- type infections [23]. However, these infections might not progress toward malignancy as Ferguson et al. reported that heterozygotic form of the HLA-G*01:01:01 allele conferred significant protection against cervical cancer [13]. In line with our results on the protective effect of allele G*01:04:01 for oral HPV infection, Metcalfe and coworkers also reported that homozygous HLA-G*01:04:01 genotype was related to a significantly decreased risk of genital HPV infection [23]..

The allele G*01:01:02 showed to reduce the probability of clearance of oral HPV-infection (OR = 0.50) and its combined genotype G*01:01:02/01:04:01 to increase oral HPV persistence (OR = 4.01). Protective effect against multiple-type infections was also noted with genotype G*01:01:01/01:01:02 (OR = 0.49) but not with the allele G*01:01:02. Our previous findings showed HLA-G*01:01:02/01:01:02 genotype concordance, between the mother and her child, to increase the risk of oral infection of the child by any HPV genotype and/or HR-HPV genotypes (OR 2.45) [29]. Ferguson et al. found significant associations between persistent genital HPV-16 and LR-HPV- infection with allele G*01:01:02, G*01:01:03, G*01:01:05, G*01:01:08 and G*01:03 (OR’s 1.90, 2.07, 2.52, 2.17, 2.99, respectively) [12]. Later, they also reported that the homozygous G*01:01:02 genotype increased the risk of developing invasive cervical squamous cell carcinoma (OR = 3.52) [13]. Contradictory to the later, Metcalfe et al. observed that allele G*01:01:02 was significantly associated with decreased risk of any genital HPV infection (OR = 0.64) and had a protective effect against multiple type infections (OR = 0.45) [23]. Regarding to allele G*01:01:03, which Ferguson et al. found to increase the risk persistent genital HPV infections (OR = 2.07) [12], we also observed this allele to increase the risk for multiple-type oral infections (OR = 3.32) but not for genital infections (Table 1).

There seems to be some parallel trends recorded in both genital and oral infections, but interestingly, in the present study we were unsuccessful to show any association between HLA-G polymorphism and genital HPV infections. The potential reasons might be that the previous studies have been somewhat larger (*N* = 539–636) [12, 13, 23], and importantly, focused on populations of different origin (Hispanics, Inuites) [23, 30], thus representing a larger scale of HLA-G polymorphism available as potential predictors of genital HPV infection outcomes, which might explain the divergent results [12, 13, 23, 30].

Only a few studies have assessed the associations between HLA-G polymorphism and female reproductive health [31, 32]. HLA-G seems to play a major role as a suppressor of the immune response at the maternal-fetal interface as well as in placental angiogenesis [33]. Craenmehr et al. reported HLA-G over-expression in the full-term placenta of the women with a history of recurrent miscarriages (OR = 6.67) [31]. In another study, HLA-G gene alleles *0106, *010106, *01010106 and *0105 N were significantly higher in patients with embryonic implantation failure on infertility treatments [32]. In the present study, we could not confirm these results on HLA-G alleles, but we showed an association with HLA-G G*01:01:02/01:04:01 genotype and risk of both infertility and undergoing treatments for infertility, OR = 5.06 and OR = 9.07, respectively (Table 4). In addition, genotype G*01:01:01/01:01:03 was significantly associated with an abnormal placenta (OR = 5.51).

This study has some potential limitations. The FFHPV cohort consists only of Caucasian Finnish women, and accordingly, the generalization to other populations is limited because Finland has its limited gene pool [34]. Cohort size of women was quite small, only 306 women, thus impeding the power to evaluate any associations of the rarest HLA-G genotypes. Regarding our reproductive risk factors all women in our cohort were pregnant at study baseline and the cohort is not a representative series of infertility clinic patients. The data on their reproductive health are based on the women’s self-reported statements. The main strength of this study is the vast database from a long-term prospective study that included study subjects with similar lifestyle and biological background, followed-up a for a long period of time with detailed HPV status of both the genital and oral sites.

## Conclusions

In conclusion, we identified six clinically significant HLA-G genotypes in women which affecting, however, only the HPV status and infection outcomes in oral mucosa, but not in genital tract. In addition, HLA-G genotype *01:01:02/01:04:01 showed to be associated with the reported history of infertility. Further studies on HLA-G polymorphism are warranted to confirm their impact as predictors of the natural history of HPV infections at different anatomic sites.

## Data Availability

Data is available upon reasonable request from the corresponding author.
